# Serum uric acid levels in patients with amyotrophic lateral sclerosis: a meta-analysis

**DOI:** 10.1038/s41598-018-19609-2

**Published:** 2018-01-18

**Authors:** Fan Zhang, Qin Zhang, Yaqiong Ke, Jianbo Hao, Ling Lu, Nannan Lu, Xiling Chen

**Affiliations:** Department of Geriatrics, The Second Affiliated Hospital of Zhengzhou University, Zhengzhou, 450014 China

## Abstract

The pathogenic mechanism of ALS remains unclear. However, increasing evidence has indicated that uric acid (UA) may play a protective role in the pathogenesis of ALS. The aim of this study was to evaluate the association between serum UA levels and ALS. A comprehensive literature search in PubMed, Embase, Web of Science, and Cochrane Library was conducted up to 31st August, 2017, using keywords. A random-effects model or fixed-effects model was used to calculate the pooled estimate according to the inter-group heterogeneity. Finally, we indentified 8 case-control and 3 cohort studies. The results indicated that patients with ALS had significant decreased levels of serum UA compared to healthy controls (standardized mean difference (SMD) = −0.72, 95% CI [−0.98,−0.46], P < 0.001). Increased serum UA levels were associated with lower all-cause mortality risk among ALS patients (risk ratio (RR) = 0.70, 95% CI [0.57, 0.87], P = 0.001). To summarize, there is an inverse association between serum UA levels and risk of death among ALS patients. Randomized controlled trials with high quality are required to elucidate the role of UA on ALS.

## Introduction

Amyotrophic lateral sclerosis (ALS) is an idiopathic, fatal neurodegenerative disease that is characterized by the degeneration of upper and lower motor neurons^1,2^. Its incidence rate is nearly 2 and 0.8 per 100,000 people-years in Western countries and East Asia, respectively^3^. The average survival time of ALS patients is approximately 2 to 5 years after symptom onset because of respiratory failure^4^. The pathogenic mechanism of ALS is still elusive. However, central nervous system seems to be especially vulnerable to oxidative stress and increasing evidence supports the theory that oxidative stress plays a crucial role in the pathogenesis of ALS^5,6^.

Uric acid (UA), which is one of the metabolic products of purine, is a natural antioxidant. UA has been hypothesized to reduce oxidative stress by scavenging reactive oxygen radicals and other reactive species^7,8^. While there is growing evidence to support a possible protective role of UA, a cause-effect relationship between UA and disease outcomes has not been firmly established yet^9–11^. Thus, we conducted this study to comprehensively assess the association between UA and ALS by integrating all available data.

## Results

### Characteristics of the included studies

**Figure 1:**
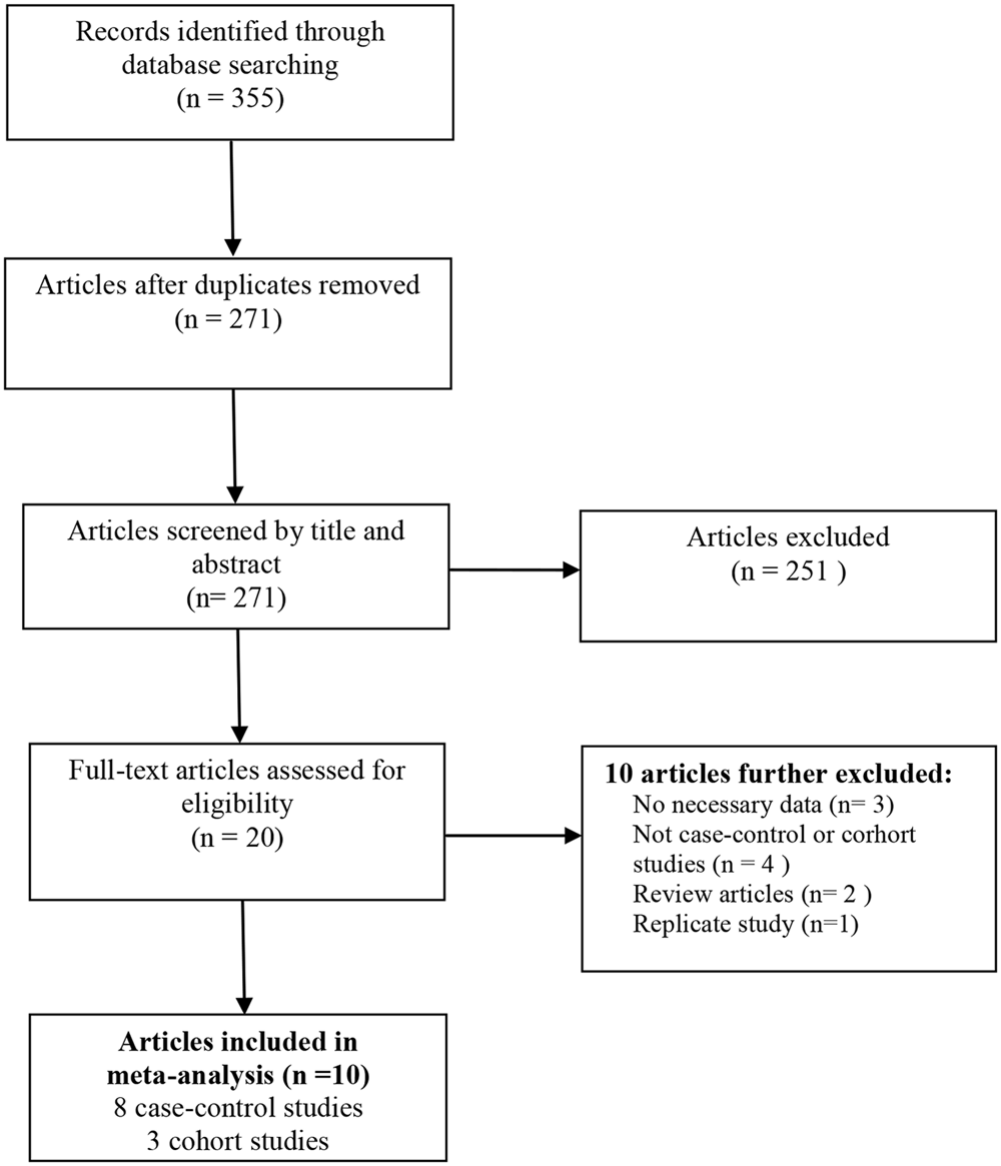
Flow chat of the study selection.

The literature research identified 355 papers from computerized databases. Among these articles, 84 were removed because they were duplicate publications. After reviewing the titles and abstracts, 251 citations were excluded for various reasons, and 20 articles were evaluated through a full-text review. Ten articles were further excluded for not meeting the inclusion criteria. Therefore, 8 case-control studies and 3 cohort studies were identified in this study^12–21^. The flow chat is shown in Fig. 1. Those 8 case-control studies included 1168 patients with ALS and 1391 healthy controls, and those 3 cohort studies included 3190 ALS patients^12–21^. Among the included studies, 7 were performed in Asians, 3 in Causations, 1 in both Asians and Causations. Most case-control studies controlled for some conventional risk factors, including age (n = 8), and gender (n = 8). However, only 3 case-control studies controlled for BMI. Those cohort studies adjusted for several variables, including age (n = 3), gender (n = 3), and BMI (n = 3). In addition, two cohort studies adjusted for disease duration. The follow-up time ranged from 13 to 21.3 months. The detailed characteristics of the included studies are presented in Tables S1 and S2. Quality assessment was performed according to the NOS criteria. The NOS score ranged from 6 to 8 points in these studies, suggesting that these studies were of moderate to high quality.

### Comparing UA levels in ALS patients and control subjects

**Figure 2:**
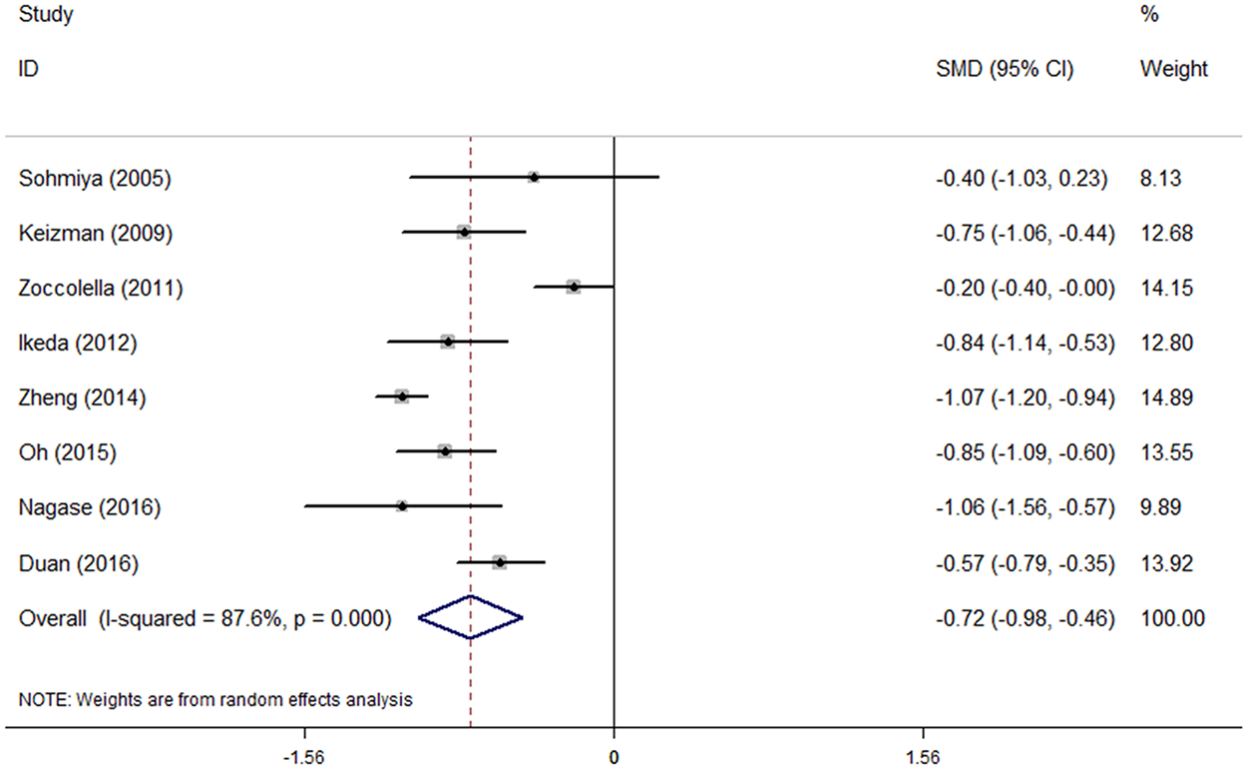
Patients with ALS had lower levels of uric acid than healthy controls.

There was substantial heterogeneity between those case-control studies (P < 0.001; I² = 87.6%). Thus, a random-effects model was used. The mean concentration of serum uric acid was lower in ALS patients compared to control subjects (SMD = −0.72, 95% CI [− 0.98, − 0.46], P < 0.001). The forest plot was shown in Fig. 2. Sensitivity analysis demonstrated that the pooled SMD was stable after omitting each study, which suggested that the results were reliable and robust (Fig. S1).

### Serum UA levels and all-cause mortality among ALS patients

**Figure 3:**
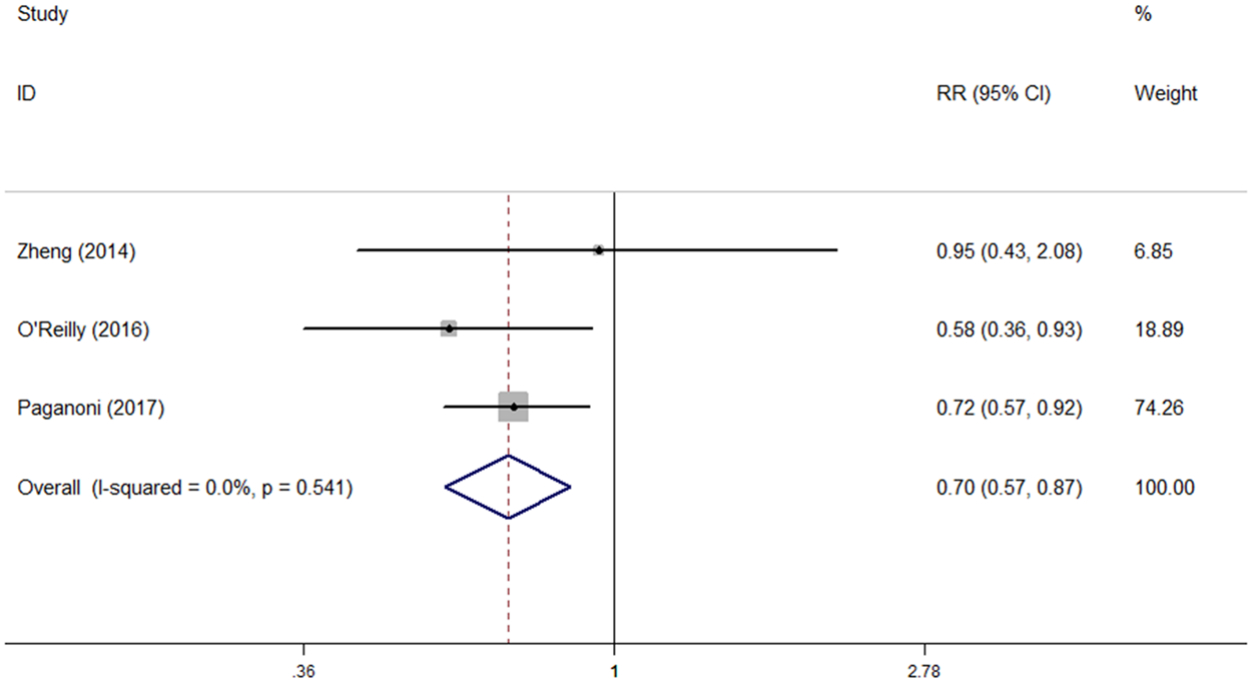
High serum uric acid levels were significantly associated with decreased risk of death among ALS patients.

Heterogeneity analysis suggested that there was no obvious heterogeneity between those cohort studies, and a fixed-effects model was used to pool the risk estimate. The results suggested that higher serum UA levels were associated with decreased all-cause mortality among ALS patients (top vs. bottom tertile RR = 0.70, 95% CI [0.57, 0.87], P = 0.001). The forest plot was shown in Fig. 3. Sensitivity analysis indicated that the pooled RR was stable after omitting each study (Fig. S2).

### Publication bias

**Figure 4:**
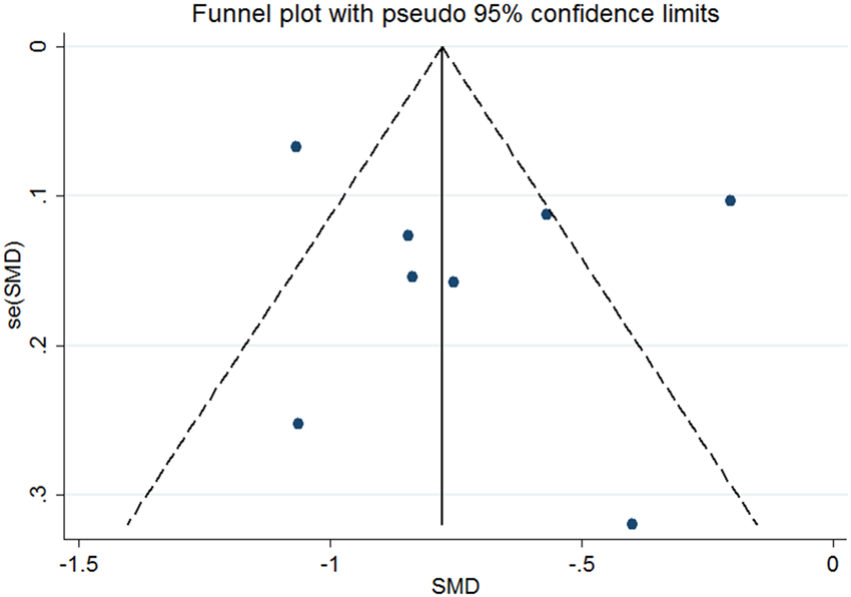
Publication bias assessment of the meta-analysis.

Publication bias was assessed by funnel plot and Egger’s test. The funnel plot showed that there was no obvious asymmetry (Fig. 4). In addition, Egger’s test did not indicate significant publication bias (P = 0.506).

## Discussion

It is important to explore the association between UA and ALS. The present meta-analysis included both case-control studies comparing the serum UA levels in ALS patients and control subjects, and cohort studies assessing the risk of death among ALS patients with different categories of serum UA levels. Our results identified that the level of serum UA in ALS patients was significantly lower than that in control subjects. We also demonstrated that elevated serum UA levels were associated with lower all-cause mortality risk in patients with ALS. The top tertile of UA levels was associated with 30% lower risk of death compared to the bottom tertile among ALS patients. Therefore, there is an inverse association between serum UA levels and risk of death among ALS patients, and UA plays a protective role in ALS. Considering the protective effect of UA, elevated UA levels could be a potential therapeutic method for ALS, and randomized controlled trails are needed.

A previous meta-analysis, which included 3 studies, found that ALS was associated with a lower concentration of serum uric acid^22^. There were some differences when compared these two meta-analyses. First, we included cohort studies assessing the all-cause mortality risk among ALS patients with different serum UA levels, which could help us assess the effect of UA levels on the risk of death among ALS patients. Second, we conducted a comprehensive search and included more recent studies (8 case-control studies and 3 cohort studies). Third, study quality was strictly evaluated, and robustness of the results was examined in this meta-analysis.

Previous epidemiological studies proved that decreased uric acid levels were associated with an increased risk of PD and AD^23,24^. UA has the ability to reduce oxidative stress, to inhibit glutamate toxicity, and to protect central nervous system^8,25^. ALS could benefit from these properties. First, superoxide, like reactive oxygen radicals, could increase oxidative neurotoxicity to neurons^26^.UA can play a protective role by removing it^27^. Second, UA could also chelate copper and iron, which meant that they cannot catalyze free-radical reactions^28^. Third, glutamate toxicity involved in the pathogenesis of ALS^29,30^. UA could reduce damage to neurons elicited by reducing glutamate toxicity^31^.

Of course, there were several limitations in this meta-analysis, so the results should be treated cautiously. First, this meta-analysis only included studies published in English and Chinese, which could inevitably lead to language bias. Second, diet and lifestyle differences might influence the metabolism of UA, while these differences could not be considered in this study. Third, selection bias might have been introduced because we fail to obtain complete data from some studies. Fourth, it has been proved that UA levels were correlated with BMI. But several included studies did not adjusted BMI between ALS patients and healthy controls, which might reduce the reliability of the study results. Fifth, we failed to conduct the dose-response analysis between serum UA levels and all-cause mortality risk among ALS patients because of limited data.

In conclusion, there is an inverse association between serum UA levels and risk of death among ALS patients. UA plays a protective role in ALS. Well-designed randomized controlled trials are required to assess the therapeutic effect of UA on ALS.

## Methods

This study was conducted according to the “Preferred Reporting Items for Systematic Reviews and Meta-analyses” (PRISMA) guidelines^32^.

### Research strategies

Four main computerized databases, including PubMed, Web of Science, Embase and Cochrane Library were searched to collect studies regarding the association between UA levels and ALS before 31st August, 2017. The research language was restricted to English and Chinese. The following search terms were used: “uric acid” or “urate”, or “urine acid”, or “UA”, and “amyotrophic lateral sclerosis” or “ALS”. Furthermore, reference lists of relevant articles were evaluated to collect all eligible articles.

### Inclusion and exclusion criteria

The inclusion criteria were as follows: (1) having definite diagnostic criteria for ALS; (2) case-control studies or cohort studies evaluating the association between serum UA levels and ALS; (3) comparing the serum UA levels in ALS patients and control subjects or assessing the all-cause mortality risk among ALS patients with different categories of serum UA levels; (4) odds risk(OR), risk ratio (RR), or Hazard risk (HR) with 95% confidence interval (CI) were reported in cohort studies, or mean value of serum UA levels with standard derivation (SD) was reported in case-control studies. The exclusion criteria were as follows: (1) necessary data were not available; (2) duplicate reports; and (3) animal studies.

### Data extraction and quality assessment

The following data were extracted from each included studies: author name, publication year, country of origin, gender, controlled factors, mean value and SD of serum UA levels, sample size of ALS cases and healthy controls, risk estimates of all-cause mortality in ALS cohorts, and adjusted confounders. Study quality was assessed by the Newcastle-Ottawa Scale (NOS)^33^. The maximum point was 9. A score of 7 or above was considered high quality. The literature search and data extraction were completed by two investigators separately. The disagreements between investigators were resolved by discussion.

### Data analysis

To compare serum UA levels in ALS patients and control subjects, standardized mean difference (SMD) with 95% CI was used. To evaluate the association between serum UA levels and all-cause mortality risk in ALS patients, the RR with 95% CI was used. The UA concentration was converted from μmol/L to mg/dl using a ratio of 16.81 (1μmol/L = 0.01681 mg/dl). HRs and ORs were assumed equally to RR. Risk estimates for UA were differently reported by individual studies (e.g., halves, tertiles, or per unit change), and thus transformed as previously described^34,35^. Briefly, log risk estimates were transformed with the comparison of top and bottom tertiles being equivalent to 1.37 times the log risk ratio for comparison between the top and bottom halves, and to 2.18 times the log risk ratio for 1-standard deviation (SD) increase.

Heterogeneity analysis was assessed using the Cochrane Q test and I² statistic^36^. If I² < 50% and P > 0.1 in the Q test, the studies were not obviously heterogeneous, and the fixed-effects model was used to calculate the pooled estimate. Otherwise, the random-effects model was used when there was substantial heterogeneity. Publication bias was assessed by funnel plot and Egger’s test^37^. To evaluate the influence of each individual study on the pooled estimate, sensitivity analysis was conducted by omitting each study by turns. All data were analyzed using the STATA12.0 software. P < 0.05 was considered statistically significant.

### Data availability

All data generated or analysed during this study are included in this article and its supplementary materials.

## Electronic supplementary material

Supplementary Information
