# Supplementary material for: Serum uric acid levels in patients with amyotrophic lateral sclerosis: a meta-analysis
Source: Sci Rep. 2018 Jan 18;8:1100. doi: 10.1038/s41598-018-19609-2 (PMC5773600; doi:10.1038/s41598-018-19609-2)
Supplement: Supplementary file 1 — Supplementary Information [file 41598_2018_19609_MOESM1_ESM.pdf]

Serum uric acid levels in patients with amyotrophic lateral sclerosis: a meta-analysis

Fan Zhang[1], Qin Zhang[1], Yaqiong Ke[1], Jianbo Hao[1], Ling Lu[1], Nannan Lu[1] & Xiling Chen[2*]

[1.] Department of Geriatrics, The Second Affiliated Hospital of Zhengzhou University, Zhengzhou 450014, China

[2.] Department of Geriatrics, The Second Affiliated Hospital of Zhengzhou University, Zhengzhou 450014, China. E-mail: chenxiling412@126.com

[*] Corresponding author: Xiling Chen, E-mail: chenxiling412@126.com, Tel/Fax: +86 371 63620506

Supplementary materials

Table S1. Characteristics of the included case-control studies

Table S2. Characteristics of the included cohort studies

Fig S1. Sensitivity analysis for the pooled SMD

Fig S2. Sensitivity analysis for the pooled RR

Table S1. Characteristics of included case-control studies

First author	Year	Study location	ALS definition	ALS		Control		Variables controlled	NOS score
				N	UA (Mean±SD)	N	UA (Mean±SD)		
Sohmiya	2005	Japan	EEC	20	266±69μmol/L	20	298±80μmol/L	Age and gender	6
Keiman	2009	Israel	EEC	86	4.78±1.3mg/dl	86	5.76±1.3 mg/dl	Age, gender, BMI, smoking, cardiovascular risk, chronic disease, and medications	7
Zoccolella	2011	Italy	EEC and REEC	132	4.5±1.48 mg/dl	337	4.8±1.48 mg/dl	Age and gender	8
Ikeda	2012	Japan	REEC	92	4.1±1.1 mg/dl	92	5.2±1.5 mg/dl	Age, gender, and BMI	6
Zheng	2014	China	REEC	512	5.0±1.5 mg/dl	501	6.5±1.3 mg/dl	Age and gender	7
Oh	2015	Korea	REEC	136	4.50±1.17 mg/dl	136	5.51±1.22 mg/dl	Age, gender and BMI	7
Nagase	2016	Japan	REEC	26	215±94μmol/L	55	314±93μmol/L	Age and gender	6
Duan	2016	China	REEC	164	310±88μmol/L	164	356±78μmol/L	Age and gender	7

ALS=Amyotrophic lateral sclerosis; EEC=El Escorial criteria; EECR=Revised El Escorial criteria; UA=Uric acid; SD=standard derivation; BMI=Body mass index; NOS= Newcastle-Ottawa Scale

Table S2. Characteristics of the included cohort studies

First author	Year	Study location	ALS definition	Duration of follow-up (months)	Study size	UA comparison	Adjusted variables	NOS score
Zheng	2014	China	REEC	21.3	512	Highest quartile vs. lowest quartile (>7.6mg/dl vs. <5.6 umol/l)	Age at onset, gender, disease duration, and BMI	8
O'Reilly	2016	Europe, North America, and Australia,	EEC	12	941	High halves vs. low halves (>5.1mg/dl vs. < 5.1mg/dl)	Age, gender, ALSFRS-R, disease duration, riluzole use, the site of onset, and BMI	7
Paganoni	2017	Europe, USA, and Japan	EEC and REEC	19	1736	HR for per unit increase	Age, gender, diagnostic delay, creatinine, and BMI	8

ALS=Amyotrophic lateral sclerosis; EEC=El Escorial criteria; EECR=Revised El Escorial criteria; UA=Uric acid; HR=hazard ratio; ALSFRS-R=ALS Functional Rating Scale-Revised; BMI=Body mass index; NOS= Newcastle-Ottawa Scale

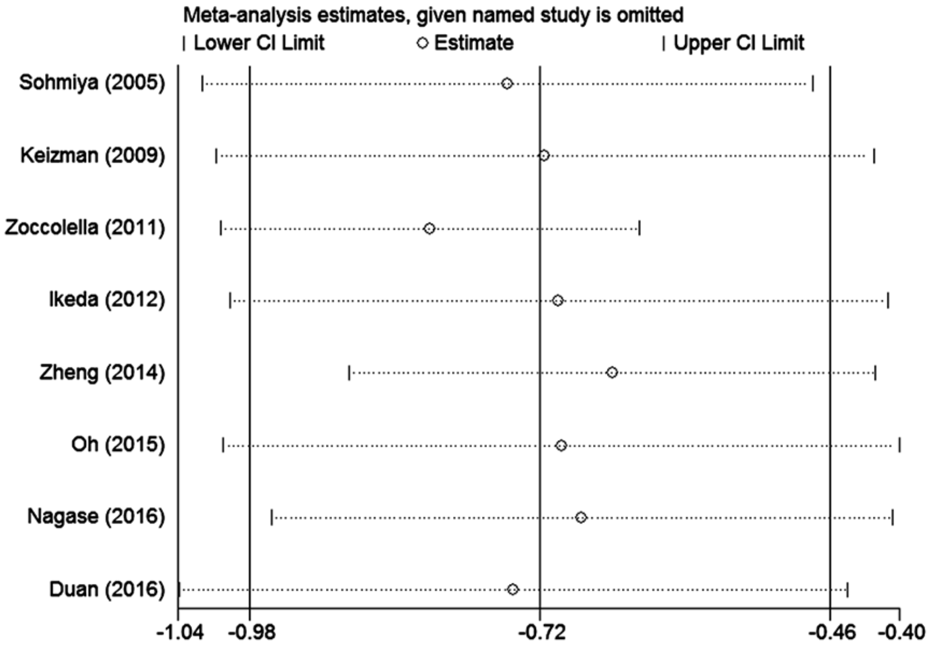

Fig S1. Sensitivity analysis for the pooled SMD

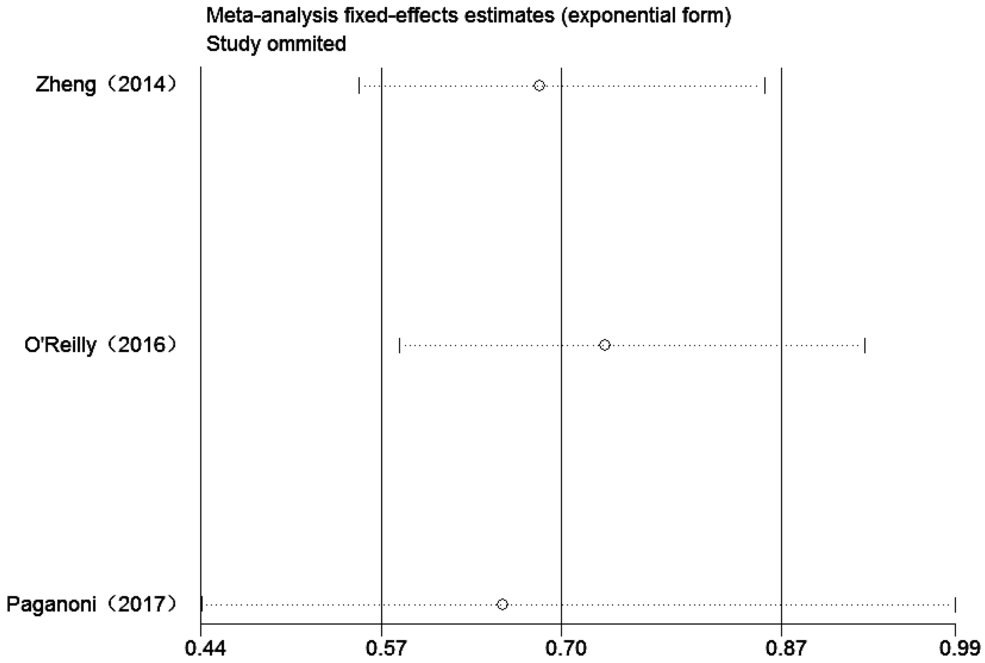

Fig S2. Sensitivity analysis for the pooled RR
